# Transcriptome analysis uncovers *Arabidopsis F-BOX STRESS INDUCED 1* as a regulator of jasmonic acid and abscisic acid stress gene expression

**DOI:** 10.1186/s12864-017-3864-6

**Published:** 2017-07-17

**Authors:** Lauren E. Gonzalez, Kristen Keller, Karen X. Chan, Megan M. Gessel, Bryan C. Thines

**Affiliations:** 1 Keck Science Department, Claremont McKenna, Pitzer, and Scripps Colleges, Claremont, CA 91711 USA; 2 Present address: Department of Genetics, Yale University School of Medicine, New Haven, CT 06510 USA; 3 Present address: Department of Biostatistics, UCLA Fielding School of Public Health, Los Angeles, CA 90095 USA; 4 Chemistry Department, University of Puget Sound, Tacoma, WA 98416 USA; 5 Biology Department, University of Puget Sound, Tacoma, WA 98416 USA

**Keywords:** Jasmonic acid, Abscisic acid, F-box, Plant stress

## Abstract

**Background:**

The ubiquitin 26S proteasome system (UPS) selectively degrades cellular proteins, which results in physiological changes to eukaryotic cells. F-box proteins are substrate adaptors within the UPS and are responsible for the diversity of potential protein targets. Plant genomes are enriched in F-box genes, but the vast majority of these have unknown roles. This work investigated the *Arabidopsis* F-box gene *F-BOX STRESS INDUCED 1* (*FBS1*) for its effects on gene expression in order elucidate its previously unknown biological function.

**Results:**

Using publically available Affymetrix ATH1 microarray data, we show that *FBS1* is significantly co-expressed in abiotic stresses with other well-characterized stress response genes, including important stress-related transcriptional regulators. This gene suite is most highly expressed in roots under cold and salt stresses. Transcriptome analysis of *fbs1–1* knock-out plants grown at a chilling temperature shows that hundreds of genes require *FBS1* for appropriate expression, and that these genes are enriched in those having roles in both abiotic and biotic stress responses. Based on both this genome-wide expression data set and quantitative real-time PCR (qPCR) analysis, it is apparent that *FBS1* is required for elevated expression of many jasmonic acid (JA) genes that have established roles in combatting environmental stresses, and that it also controls a subset of JA biosynthesis genes. *FBS1* also significantly impacts abscisic acid (ABA) regulated genes, but this interaction is more complex, as *FBS1* has both positive and negative effects on ABA-inducible and ABA-repressible gene modules. One noteworthy effect of *FBS1* on ABA-related stress processes, however, is the restraint it imposes on the expression of multiple class I *LIPID TRANSFER PROTEIN* (*LTP*) gene family members that have demonstrated protective effects in water deficit-related stresses.

**Conclusion:**

*FBS1* impacts plant stress responses by regulating hundreds of genes that respond to the plant stress hormones JA and ABA. The positive effect that *FBS1* has on JA processes and the negative effect it has on at least some ABA processes indicates that it in part regulates cellular responses balanced between these two important stress hormones. More broadly then, *FBS1* may aid plant cells in switching between certain biotic (JA) and abiotic (ABA) stress responses. Finally, because *FBS1* regulates a subset of JA biosynthesis and response genes, we conclude that it might have a role in tuning hormone responses to particular circumstances at the transcriptional level.

**Electronic supplementary material:**

The online version of this article (doi:10.1186/s12864-017-3864-6) contains supplementary material, which is available to authorized users.

## Background

The ubiquitin 26S proteasome system (UPS) coordinates the selective degradation of proteins, leading to important intracellular physiological changes [1]. Specific proteins destined for removal by the UPS are marked by covalent attachment of ubiquitin, which signals for recognition and degradation by the 26S proteasome. A three-part enzyme cascade accomplishes ubiquitylation, in which the last component, an E3 ubiquitin ligase, specifically interacts with and ubiquitylates the target [2]. It is estimated that the plant *Arabidopsis thaliana* can assemble over 1500 different E3 enzyme complexes, each of which may target multiple proteins in the same family [2]. Thus, the impact of E3 ligases in sculpting plant cellular proteomes is far-reaching. While numerous types of E3 ligases exist, a prevalent type in plants is the Skp1-Cullin-F-box (SCF) complex [3, 4]. Skp1 and Cullin proteins, along with an Rbx protein, form the common core part of all SCF complexes [5–8]. However, F-box proteins vary from complex to complex and act as substrate adaptors, each interacting with and recruiting unique targets [3, 9, 10]. Therefore, F-box proteins are a critical connection between the UPS and cellular effects. Well-studied plant F-box proteins have pivotal roles in diverse physiological processes, including hormone perception and signaling, development, reproduction, defense, light perception, and the circadian clock [11–18].

Plant genomes examined to date are enriched in F-box genes by about an order of magnitude compared to many non-plant genomes [2, 3]. There are 698, 764, 359, and 972 F-box genes in *Arabidopsis*, rice, maize, and *Medicago truncatula*, but only 20, 27, and 69 in *Saccharomyces*, *Drosophila*, and humans, respectively [2–4, 19]. As approximately 700 of the 27,000 predicted *Arabidopsis thaliana* genes encode F-box proteins, it is one of the largest gene families in this model species; however, less than 15% of these F-box genes have been assigned certain biological function. In this context, the F-box gene family is a largely unexplored but fundamentally important aspect of plant biology. Exact reasons for this F-box gene enrichment in plants are enigmatic, but it almost certainly reflects an extensive repertoire of response mechanisms that plants, as sessile organisms, use to combat environmental challenges. Genome-scale studies bolster the notion that SCF complexes are broadly important to plant stress responses. For example, 83% of F-box genes in chickpea are differentially expressed in at least one stress condition [20].

Expression-based investigations can suggest important but previously unrecognized roles for F-box genes in various abiotic and biotic stress responses. In both *Arabidopsis thaliana* and *Phaseolus vulgaris*, the F-box gene *F-BOX STRESS INDUCED1* (*FBS1*) is rapidly induced by unfavorable environmental conditions, such as salt and osmotic stress, and also by wounding and the bacterial pathogen *Psuedomonas syringae* [21]. *FBS1* expression is also increased by treatment with the major plant stress hormones jasmonic acid (JA), ethylene, salicylic acid (SA), and abscisic acid (ABA) [21]. This broad induction profile of *FBS1* suggests that it may fulfill an important cellular function common to many diverse stress responses. However, neither the ubiquitylation targets of SCF^FBS1^ nor the phenotype of *fbs1* loss-of-function plants are established, so the connection between *FBS1* and specific stress response networks is unclear.

We sought to understand how *FBS1* works in stress gene networks by investigating genes that depend upon *FBS1* for normal expression. Here, we show that *FBS1* belongs to a group of important transcriptional regulators and other signaling genes that act in salt, drought, and cold responses, as well as in responses to pathogens. Transcriptome analysis demonstrates that *FBS1* function influences a number of important stress responsive processes and, in particular, that it regulates genes responding to the stress hormones jasmonic acid (JA) and abscisic acid (ABA). Additionally, *FBS1* also affects the expression of a subset of JA biosynthesis genes. These findings suggest that *FBS1* likely impacts significant effects of important stress hormones, which are required for plant responses in unfavorable environmental conditions.

## Results

### Abiotic stress expression profiles of *FBS1* and co-expressed genes

**Fig. 1:**
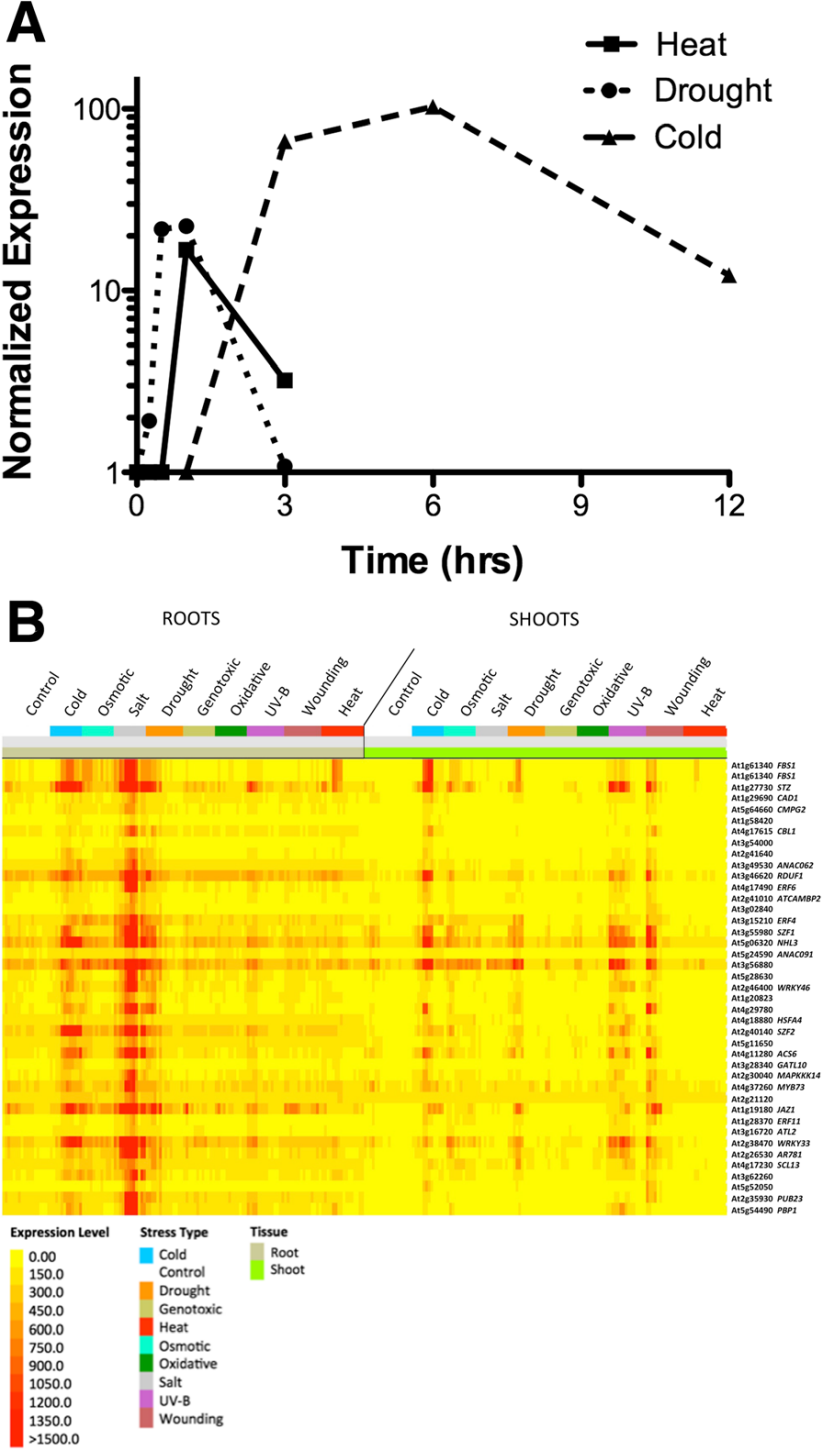
Expression profiles of *FBS1* and co-expressed genes in abiotic stresses. **a** Time course analysis of *FBS1* expression in AtGenExpress ATH1 array data sets from heat, drought, and cold treated 18 day-old *Arabidopsis* plants. The average of the two available biological replicates is shown; all time points are normalized to time 0 (untreated). **b** Thirty-nine genes with expression patterns significantly correlating (*r* > 0.75) with *FBS1* expression*.* Shown is absolute expression across 272 ATH1 array samples from 16 day-old *Arabidopsis* roots and shoots that experienced one of nine different abiotic stresses or control conditions. For each treatment, the exposure time for the given stress increases from left to right

To identify F-box genes in the *Arabidopsis* genome with possible abiotic stress response roles, we analyzed publically available Affymetrix ATH1 array stress data sets for F-box genes induced or repressed in the 24 h after onset of the stress condition. One F-box gene in particular, *FBS1* (At1g61340), was induced by multiple stresses, including drought, heat, and cold stresses (Fig. 1a). Both drought and heat (38 °C) induced *FBS1* over 10-fold within the first hour of stress treatment in 18 day-old plants, while expression declined in both stress conditions by three hours (Fig. 1a). In contrast, exposure of 18 day-old plants to 4 °C induced *FBS1* approximately 60-fold and 100-fold at three and six hours, respectively, with expression declining by 12 h (Fig. 1a). *FBS1* expression in *Arabidopsis* seedlings was independently validated by qPCR for heat stress, and results were similar both in timing and magnitude of induction to the expression pattern measured with the arrays (Additional file 1: Figure S1). Consistent with the stress induction profiles that we observed in this array data, *FBS1* was previously shown to be inducible by wounding, osmotic, and salt stresses, as well as by *Pseudomonas* infection and numerous plant stress hormones [21]. Collectively, these findings show that *FBS1* is induced by a wide range of both biotic and abiotic stresses, and suggests that this gene could be an important common component of responses to different stresses.

To gain support for the hypothesis that *FBS1* is important for stress responses and to obtain further clues regarding its function, we searched for genes represented on the Affymetrix ATH1 array with similar expression profiles to *FBS1* across various abiotic stress conditions in roots and shoots in 16 to 18 day-old plants using Expression Angler from the Bio-Analytic Resource (BAR) [22]. Thirty-nine genes had expression profiles similar to *FBS1* across nine abiotic stress conditions (Pearson correlation coefficient, *r* > 0.75) (Fig. 1b). Cold, salt (NaCl), and drought treatments were the most potent inducers of *FBS1* and co-expressed genes in root tissue (Fig. 1b, left side), while cold, drought, UV-B light, and wounding induced the highest levels of *FBS1* and co-expressed genes in shoots (Fig. 1b, right side). *FBS1* and co-expressed genes were, in general, more highly stress-inducible in roots than in shoots, which may reflect a more important role for *FBS1* in biological processes, particularly cold and salt responses, in this organ.

Notably, 24 of the 39 genes co-expressed with *FBS1* (Fig. 1b) have been experimentally validated as having a defined role in biotic and/or abiotic stress responses (Additional file 6: Table S1). Sixteen of these 24 genes mitigate the effects of cold, reactive oxygen species (ROS), salt, or other osmotic stresses, while ten have been linked to pathogen responses or plant immunity (Additional file 6: Table S1). Significantly, 17 of the 24 validated stress genes are signaling components or transcriptional regulators of stress-responsive gene expression networks, and many of these appear to be master regulators or are otherwise central to signaling events. Some of these transcriptional regulators inhibit stress responses (*STZ*, *SZF1*, *SZF2*, *WRKY46*, *WRKY33*, *JAZ1, MYB73)*, while others act positively (*ATCBL1*, *NAC062*, *HSFA4*) [23–31]. Notably, many of these regulators directly influence the action of stress hormone pathways, including jasmonic acid (JA), abscisic acid (ABA), or ethylene. As examples, *JAZ1* is a central transcriptional repressor of jasmonic acid early response genes, *STZ* inhibits salt stress responses in part by repressing JA biosynthesis gene *LOX3*, and *ERF4* negatively regulates sensitivity to ABA and ethylene [9, 32–34]. The presence of both positive and negative regulators in this list of genes co-expressed with *FBS1* is not surprising, as stress response pathways are complex and finely tuned to specific conditions. In order to appropriately allocate resources in these energetically expensive processes, particular pathways are often activated while competing pathways are simultaneously inhibited [35]. Additionally, it is often the case that stress pathways are activated and then immediately repressed through action of a negative regulator, as hyper-activation can ultimately be detrimental to the cell [36]. The presence of *FBS1* within a set of established and important regulatory genes induced by stresses, whether they play positive or negative roles, strongly suggests that *FBS1* is part of a suite of genes that mitigates effects of adverse environmental conditions or other biotic stresses.

### The *FBS1*-dependent transcriptome at chilling temperature

We obtained an *Arabidopsis* line harboring a transposon insertion in the first intron of *FBS1*, designated *fbs1–1* hereafter, to investigate *FBS1* function. No *FBS1* transcript was detected in *fbs1–1* plants by RT-PCR. A previous study reported no obvious phenotype in salt or osmotic stress when this same insertion was part of a triple mutant, along with gene knock-outs of family members *FBS2* and *FBS3* [21]. We therefore chose to investigate the *FBS1*-dependent transcriptome in order to gain insight as to how *FBS1* affects biological processes in plant cells under stress.

Chilling treatment (4 °C) was one of the strongest inducers of *FBS1* and co-expressed genes in both roots and shoots (Fig. 1b) and so we chose a similar environmental condition to use in a comparison between *fbs1–1* and WT seedlings. Seedlings were grown at a non-stressful temperature (24 °C) for five days and then transferred to a chilling temperature (10 °C) and grown for seven additional days before gene expression was measured. The 10 °C chilling temperature effectively induced canonical cold response genes, such as C-repeat Binding Factors (*CBFs*) *1, 2,* and *3,* and it induced *FBS1* in WT (Additional file 2: Figure S2), indicating that this condition was appropriate to study the effects of chilling temperature*.* After this treatment regimen, 267 genes were more highly expressed in WT seedlings and 254 genes were more highly expressed in *fbs1–1* (Additional file 7: Table S2 and Additional file 8: Table S3). These lists represent collective differences and include: 1) downstream genes that are dependent upon *FBS1* in chilling temperature-treated plants, and 2) genes that are differentially expressed between WT and *fbs1–1* regardless of environmental condition.

**Fig. 2:**
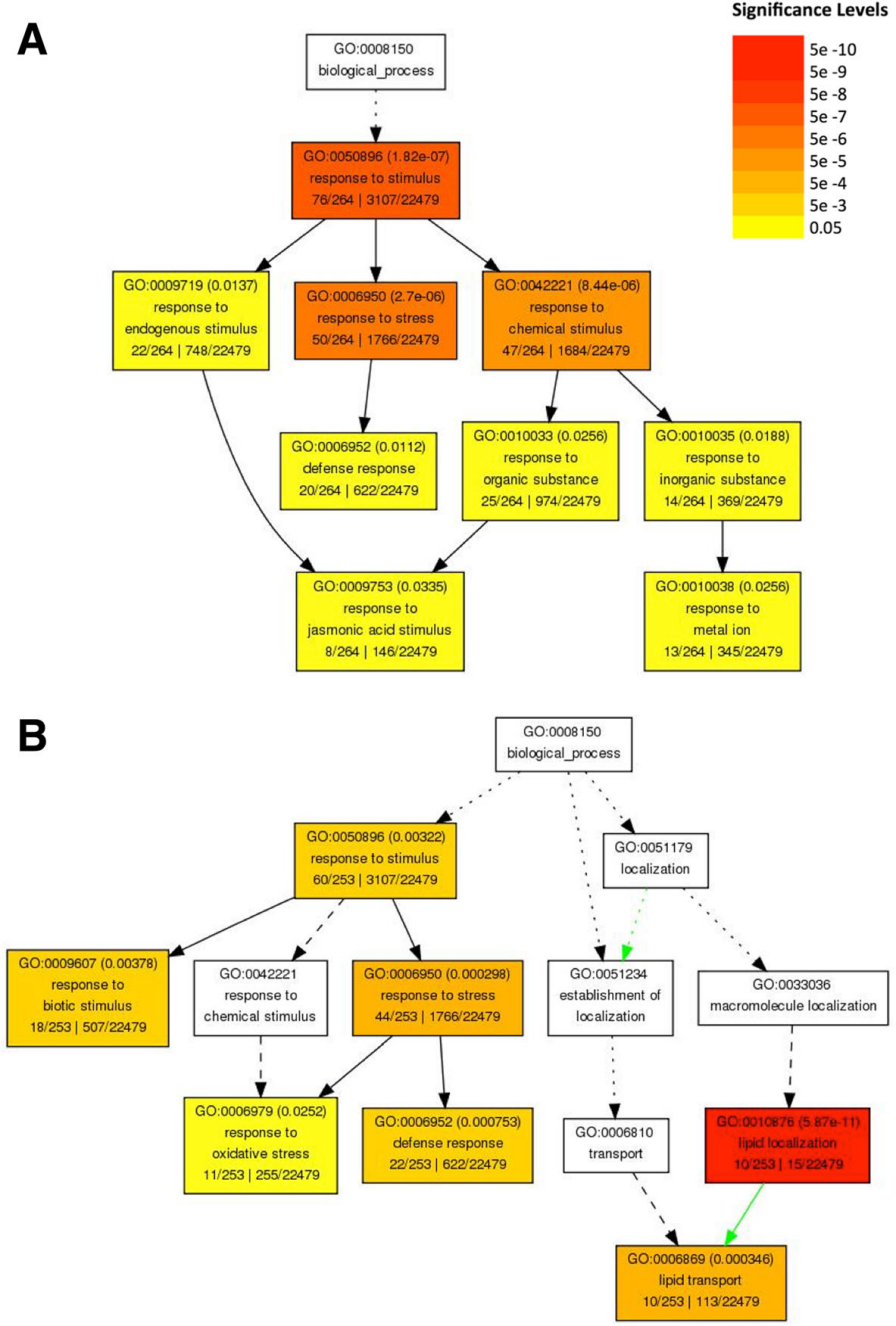
Gene Ontology (GO) term enrichment analysis for FBS1-dependent genes. Biological processes significantly enriched for genes with higher expression in WT (**a**) and genes with higher expression in *fbs1–1* seedlings (**b**) are shown. Each box indicates the GO term and description with the FDR adjusted *p*-value, and the color scale reflects these adjusted *p*-values. The fraction on the left side at the bottom is the number of genes in our dataset falling into that GO category out of the total number of genes in the list. The fraction on the right is the total number of genes on the ATH1 array falling into that GO category, out of the total number of genes represented on the array. Boxes with GO terms are presented hierarchically, with the root term at the top and child terms toward the bottom. Solid, dashed, and dotted lines show either two, one, or zero enriched terms at either end of the line; the green line indicates negative regulation

The lists of 267 and 254 differentially expressed genes between WT and *fbs1–1* were investigated for Gene Ontology (GO) term enrichment of biological processes [37]. Both gene lists were significantly enriched (False Discovery Rate < 0.05) in genes mapping to stress and defense response GO categories, which supports the hypothesis that *FBS1* impacts gene expression networks that contribute to plant survivability under adverse conditions (Fig. 2). Within the genes more highly expressed in WT, 47 mapped to the stress response category and 19 genes mapped to defense response; among genes more highly expressed in *fbs1–1*, 37 and 18 genes were assigned to these same categories, respectively. Enrichment of genes in these two stress categories is consistent with the broad stress induction profile of *FBS1* across multiple abiotic stress conditions and is consistent with the fact that many genes co-expressed with *FBS1* have roles in abiotic and biotic stresses. Furthermore, it indicates that *FBS1* may itself have a regulatory impact on stress genes and cellular stress responses. These two gene lists were also enriched in genes mapping to GO terms that relate to stress hormone processes, which suggests that *FBS1* may protect plants by altering synthesis, signaling, or subsequent events in hormone pathways. Genes more highly expressed in WT were enriched with genes involved in responses to jasmonic acid (Fig. 2a, Additional file 9: Table S4). In contrast, the genes more highly expressed in *fbs1–1* were enriched in genes acting in lipid localization and lipid transport, and specific genes from our data set assigned to this GO category regulate ABA biosynthesis (Fig. 2b, Additional file 10: Table S5).

The significant enrichment of JA response genes among all genes with elevated expression in WT is a novel link between *FBS1* and the stress hormone JA. Six of these JA genes have direct roles in transcription regulation: *JASMONATE ZIM-DOMAIN6* (*JAZ6*), *JAZ9, ETHYLENE RESPONSE FACTOR2 (ERF2)*, *MYB7*, *MYB47*, and *MYBL2* (Additional file 9: Table S4). *JAZ6* and *JAZ9* are part of a negative feedback transcription loop activated in the presence of JA [9, 32]. *JAZ* genes encode central repressors of JA-activated transcription networks, and JAZ proteins interact with and inhibit DNA-binding MYC transcription factors that directly activate JA early response genes. JAZ proteins are degraded in the presence of active JAs (ie. JA-isoleucine) to relieve restraint on JA gene networks, but most *JAZ* genes are among the first genes induced to feed back and appropriately limit pathway activation [9, 32]. That both *JAZ6* and *JAZ9* genes were expressed approximately ten-fold more highly in WT seedlings indicates that core JA signaling was more active in WT than in *fbs1–1* under our test conditions. Another important regulatory gene in this list, *ERF2*, encodes an AP2 domain transcription factor that activates expression of JA regulated defense genes and enhances resistance to the fungal pathogen *Fusarium oxysporum* [38]. The three MYB family transcription factors regulate processes central to stress responses. *MYB7 (R2R3)* is salt inducible; it represses flavonol production while increasing levels of anthocyanins, which function in UV light absorption, microbial defense, and ROS scavenging [39]. The *MYB7* promoter is bound by MYB112, which positively regulates *MYB7* expression and anthocyanin production under salt stress [40]. *MYB112* is not assigned to the JA response GO list, however, it is in our list of 267 genes more highly expressed in WT seedlings (Additional file 7: Table S2) and can therefore also be linked to JA because of its regulatory effect on *MYB7*. In contrast to *MYB7* and *MYB112*, *MYBL2* negatively regulates aspects of anthocyanin and proanthocyanidin metabolism, which may indicate activation and then subsequent restraint on stress-induced anthocyanin production [41]. The last MYB family transcription factor gene, *MYB47*, is not functionally defined, but is JA inducible and has been linked to both dehydration and cold stress responses [42]. The two additional JA response genes are *CORI3*, a tyrosine amino transferase and established JA response marker gene, and *RNS1*, a secreted ribonuclease that responds to numerous stress conditions [43, 44].

Genes with functions in lipid localization and, more specifically, lipid transport were significantly over-represented among all genes more highly expressed in *fbs1–1*. The same ten genes were assigned to these two categories, and four of these genes encode lipid transfer proteins (LTPs) (Additional file 10: Table S5). In addition, *LTP3* was more highly expressed in *fbs1–1*, though it is not assigned to this GO category (Additional file 8: Table S3). Notably, all five of these *LTPs* (*LTP2, 3, 4, 6,* and *7*) belong to the type I *LTP* gene family, which has 12 members in *Arabidopsis* [45]. In diverse plant species, including *Arabidopsis,* wheat, rice, and tree tobacco, *LTP* genes are coordinately up-regulated during dehydration-related stresses, such as drought, salt, and freezing temperatures, to protect against water loss [46–48]. Over-expression of *LTP3* in *Arabidopsis* leads to constitutive freezing tolerance, and over-expression of a rice LTP gene, *OsDIL,* results in drought tolerant rice plants [48, 49]. The increased expression of these *LTPs* in both *Arabidopsis* and rice also results in increased ABA biosynthetic activity, suggesting that at least some *LTPs* positively impact endogenous levels of this stress hormone [48, 50]. Therefore, it is plausible that *FBS1* affects water deficit related stress outcomes and some ABA-dependent processes by influencing expression of the type I *LTP* gene suite.

### Hormone effects on FBS1-dependent genes

**Fig. 3:**
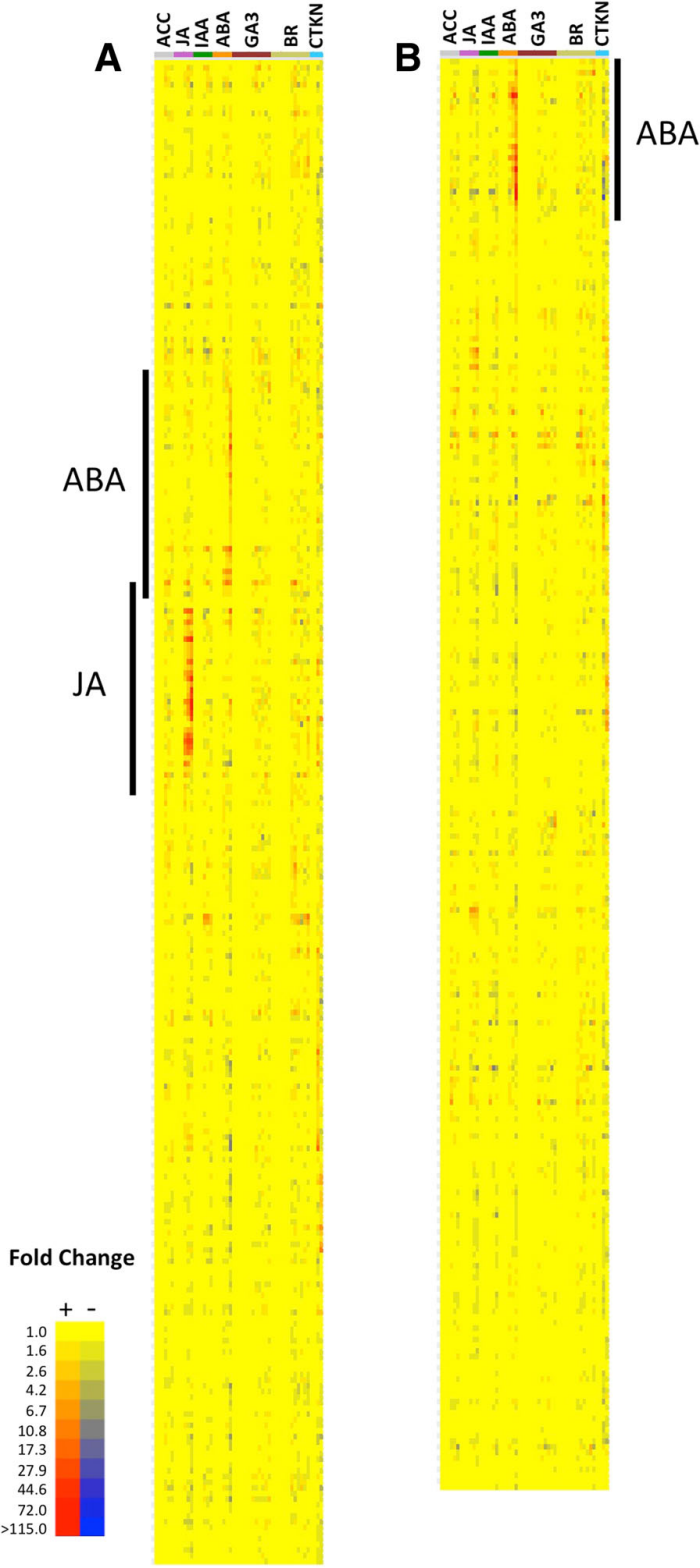
Hierarchical clustering of expression patterns in AtGenExpress hormone datasets. Shown are heat maps for (**a**) 267 genes more highly expressed in wild type and (**b**) 254 genes more highly expressed in *fbs1–1* plants. Data set abbreviations are 1-aminocyclopropane-1-carboxylic acid (ACC, ethylene), methyl jasmonate (JA), indole-3-acetic acid (IAA, auxin), abscisic acid (ABA), gibberellin (GA3), Brassinosteroids (BR), Cytokinin (CTKN). For each treatment, the exposure time for the given chemical increases from left to right. Genes are hierarchically clustered on the y-axis according to expression profile similarity

To gain more insight as to the extent of *FBS1* interaction with hormone pathways, we examined the expression patterns of the 267 and 254 genes more highly expressed in WT and *fbs1–1,* respectively, across 53 arrays representing transcript levels in hormone-treated seven day-old seedlings. In the 267 genes more highly expressed in WT, two clusters of hormone-inducible genes were most apparent: 1) methyl jasmonate (MeJA)-induced (28 genes) and 2) ABA-induced (36 genes) (Fig. 3a). Relative to ABA-induced genes, those induced by MeJA were more highly expressed during the three hours of 10 μM MeJA treatment, although some of this induction effect could be due to relative concentrations of hormones used in the treatments. However, when 68 additional hormone-related data sets were used in this analysis, the MeJA cluster was even more striking relative to all other chemical treatments and conditions, including ABA, for which array data sets were available (Additional file 3: Figure S3). This indicates that a primary positive effect of *FBS1* in WT is on JA inducible genes.

Combining the eight genes in the response to JA GO category (Fig. 2a, Additional file 9: Table S4) with these 28 JA-inducible genes (Fig. 3a) yielded a non-redundant list of 31 JA genes (Table 1). Within this collective JA gene list are core regulators of JA signaling (*JAZ6, JAZ9*), established marker genes of JA action (*CORI1*, *CORI3*), and a gene in JA biosynthesis (*AOC1*). In the 254 genes more highly expressed in *fbs1–1*, 29 ABA inducible genes formed the most apparent cluster (Fig. 3b). Although few of these 29 genes had experimental support for their biological roles, five of the ten genes in our lipid localization/transport lists (Fig. 2b, Additional file 10: Table S5) were identified by this measure as ABA-inducible. Eight other genes in this *fbs1–1* ABA cluster have putative roles in metabolism or transport, and two additional genes are established regulators of salt stress responses. Compared to JA and ABA effects, *FBS1*-dependent genes in both lists were negligibly affected by the growth hormones GA, IAA, and brassinolide, suggesting that *FBS1* does not primarily function to regulate plant growth via these hormones, at least under the experimental conditions we tested here. Instead, these results further support a role for SCF^FBS1^ as a regulator of stress responses, and these findings indicate that it acts in part by tuning a suite of JA and ABA responsive genes. The impact of *FBS1* on important JA-related genes (Table 1) and the clear impact on JA responsive genes (Fig. 3a and Additional file 3: Figure S3) led us to further consider the interaction between *FBS1* and JA.

**Table 1:** Jasmonic acid genes more highly expressed in wild type seedlings

AGI Number	Common Name	Expression After MeJA Treatment			Expression WT to *fbs1–1*	Annotation
		0.5 h	1 h	3 h		
At1g52000		3.0	12.1	34.3	5.6	Mannose-binding lectin superfamily protein
At1g52400	*BGLU18*	1.0	2.3	7.5	11.2	Beta glucosidase 18
At1g52030	*MBP2*	0.9	2.8	39.4	12.5	Myrosinase binding protein 2
At1g52410	*TSA1*	1.0	3.2	34.3	5.9	Calcium binding repeat sequence, possible role in mitosis
At3g28220		1.4	7.0	59.7	3.9	TRAF-like family protein
At4g17470		5.3	11.3	111.4	1.9	Alpha/beta-hydrolases superfamily protein
At5g24420	*PGL5*	3.0	8.0	48.5	2.9	Cytosolic 6-phosphogluconolactonase
At3g28270	*AFL1*	1.0	2.0	32.0	5.6	Peripheral membrane protein, growth regulation in drought
At3g44970		1.0	0.9	1.9	1.9	Cytochrome P450 superfamily protein
At1g18710	*MYB47*	12.1	13.9	21.1	10.6	R2R3-MYB transcription factor
At3g04000		1.1	2.5	4.3	1.9	NAD(P)-binding Rossmann-fold superfamily protein
At1g53870		1.9	2.8	3.7	1.9	Protein of unknown function
At1g44790		1.5	2.0	3.2	2.1	ChaC-like family protein
At1g19670	*CORI1*	10.6	24.3	34.3	1.9	Chlorophyllase, chlorophyll degradation
At4g21903		1.3	2.5	4.6	1.9	MATE efflux family protein, transporter
At3g25760	*AOC1*	2.0	2.8	4.0	2.6	Allene oxide cyclase I, jasmonic acid biosynthesis
At3g51450		4.3	7.5	11.3	2.4	Calcium-dependent phosphotriesterase superfamily protein
At4g23600	*CORI3*	1.4	2.0	4.3	37.5	Cystine lyase, metabolism of ethylene precursors
At1g66760		4.0	10.6	7.0	1.7	MATE efflux family protein, transporter
At4g30270	*XTH24*	1.4	2.6	1.9	2.2	xyloglucan endotransglucosylase
At1g12240	*FRUCT4*	2.8	4.0	3.5	2.4	Vacuolar invertase
At1g44350	*ILL6*	8.0	13.9	9.8	1.8	Similar to IAA amino acid conjugate hydrolase
At1g72450	*JAZ6*	8.0	9.2	6.5	2.7	Central negative regulator of jasmonic acid genes
At1g70700	*JAZ9*	13.9	29.9	26.0	1.9	Central negative regulator of jasmonic acid genes
At5g05600		7.5	19.7	17.1	3.2	2-oxoglutarate (2OG) and Fe(II)-dependent oxygenase
At3g50270		1.5	1.7	2.1	2.9	HXXXD-type acyl-transferase family protein
At5g47220	*ERF2*	9.2	6.1	1.7	2.3	Transcription factor, positive regulator of JA defense
At1g66100		1.9	8.6	15.0	116.8	Pathogenesis related protein, plant thionin
At1g71030	*MYBL2*	0.5	0.4	0.6	1.9	Transcription factor, negatively controls anthocyanin synthesis
At2g02990	*RNS1*	0.7	1.6	0.9	1.9	Ribonuclease, responds to inorganic phosphate starvation
At2g16720	*MYB7*	0.9	1.4	1.2	1.8	Transcription factor, induced by salt, inhibits flavonol production

Shown are normalized expression levels from the AtGenExpress data set for seven day-old seedlings treated for 0.5, 1, or 3 h with MeJA treatment and normalized expression levels from a comparison between WT and *fbs1–1* in 12 day-old seedlings after seven days of 10 °C treatment. *Arabidopsis* Genome Initiative (AGI) numbers, common gene names, and abbreviated annotations are based on the TAIR10 genome

### JA biosynthesis genes are mis-expressed in *fbs1–1*

**Fig. 4:**
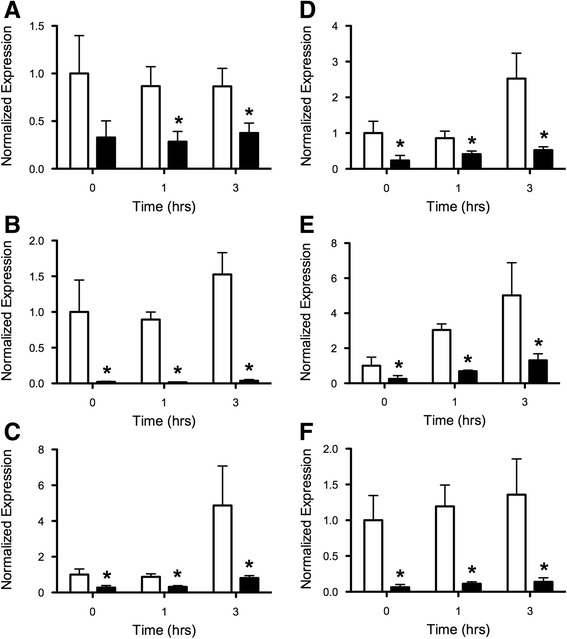
qPCR analysis of differentially expressed JA biosynthetic genes. Seven day-old *Arabidopsis* wild type (*white bars*) and *fbs1–1* (*black bars*) seedlings were either untreated (time 0) or treated for up to three hours with 10 °C chilling temperature. Transcript abundances for **a** *LOX2*, **b** *AOC1*, **c** *AOC2*, **d** *AOC3*, **e** *AOS*, and **f** *OPR1* are shown. All genes have statistically significant differences between the genotypes for at least one time point (two-way ANOVA; asterisks indicate statistical significance *p* < 0.05 between genotypes using Bonferroni post-tests). Shown are the average relative quantities (NRQs) (± SEM) normalized to *IPP2* and *PP2A* within the same sample and to the wild type expression level for that gene in untreated seedlings, which was set to 1, from three independent experimental replicates

To better understand the extent to which *FBS1* affects JA processes, we extended our analysis to genes involved in JA biosynthesis early in chilling stress. Using qPCR, we measured the expression of 14 JA biosynthesis genes belonging to the *LIPOXYGENASE* (*LOX*), *ALLENE OXIDE CYCLASE* (*AOC*), *ALLENE OXIDE SYNTHASE* (*AOS*), and *12-OXOPHYTODIENOIC ACID REDUCTASE* (*OPR*) gene families. Transcript abundances were assessed in unstressed WT and *fbs1–1* seedlings and in up to three hours of 10 °C treatment (Fig. 4a–f, Additional file 4: Figure S4). The first three hours of 10 °C temperature significantly induced the expression of three JA biosynthetic genes in WT seedlings: *AOC2*, *AOS*, and *LOX3* (Fig. 4c, d, and Additional file 4: Figure S4; two-way ANOVA, *p* < 0.05), supporting the notion that the 10 °C chilling temperature was effective in altering JA stress gene expression, as in the case of *CBFs 1–3* and *FBS1* (Additional file 2: Figure S2). Eight of the JA biosynthesis genes tested showed no statistically significant difference in expression between WT and *fbs1–1* at any point in this time course (Additional file 4: Figure S4; two-way ANOVA, *p* > 0.05). However, six JA biosynthesis genes had significantly different expression between WT and *fbs1–1* at two or more time points tested (Fig. 4a–f; two-way ANOVA, *p* < 0.05). Importantly, *ALLENE OXIDE SYNTHASE* (*AOS*) is the committed step to JA biosynthesis, and catalyzes the reaction after which all subsequent intermediates are used exclusively for JA [51]. *AOS* transcript abundance was about four times lower in *fbs1–1* relative to WT at all three time points tested (Fig. 4e), suggesting that metabolic flux through this critical step could also be reduced. The gene with the most drastic differences in expression was *AOC1* (also known as *EARLY RESPONSE TO DEHYDRATION12*), where transcript abundance in *fbs1–1* was over three orders of magnitude lower than in WT at all time points (Fig. 4b). Furthermore, all six differentially expressed genes had reduced expression even under non-stressful conditions, and any differences between the two genotypes at later time points appeared to stem from different initial transcript abundances. *AOC2*, for example, was expressed approximately five times more highly in seedlings after three hours of 10 °C treatment relative to untreated seedlings, and this degree of overall induction was nearly identical in WT (Fig. 4c). However, it appears likely that absolute *AOC2* transcript abundance at three hours did not reach a level comparable to WT because it started from a much lower initial level. Therefore, *FBS1* significantly affects expression of multiple JA biosynthesis genes, and differences observed under stress conditions could stem from differences in basal transcript abundance present in un-stressed conditions.

A simple explanation for the under-expression of downstream JA genes in *fbs1–1* (Fig. 3) could be that jasmonic acid or a bioactive derivative (ie. JA-isoleucine) is present at lower levels in this mutant compared to WT. This idea was initially attractive because *FBS1* affects expression of some JA biosynthesis genes, including *ALLENE OXIDE SYNTHASE* (*AOS)*, the enzyme catalyzing the committed step leading to JA (Fig. 4). To test whether JA levels were elevated in WT relative to *fbs1–1*, we grew seedlings in conditions identical to those used for our array analysis (Figs. 2 and 3) and, after seven days in cold or control conditions, we attempted to extract and measure total JA and JA-Ile levels by liquid chromatography-mass spectrometry (LC-MS) analysis according to established protocols [52]. However, the level of JA, if present, fell below the detection limit for this instrument (~1 pmol) in both WT and *fbs1–1* (Additional file 5: Figure S5). Similar negative results were found for JA-Ile (not shown). Thus, an extended cold treatment did not yield an obvious change in JA or JA-Ile levels at seven days that directly corresponded to our measured gene expression changes.

## Discussion

### The effect of *FBS1* on JA processes

Elucidating novel connections between F-box proteins and stress hormone pathways is essential for comprehending how plants use the UPS to endure adverse environmental conditions. This investigation shows that the F-box gene *FBS1* profoundly effects the expression of a set of genes intimately connected to JA, ABA, and other stress related processes. Loss of *FBS1* function led to a significant expression decrease in genes normally inducible by JA, including a number of important transcriptional regulators. Furthermore, six of fourteen genes examined here that are involved in JA biosynthesis also require *FBS1* for normal expression.

The lack of measurable JAs in both WT and *fbs1–1* seedlings prevented us from making definitive conclusions about whether decreased JA biosynthesis gene expression in *fbs1–1* results in reduced JA abundance. The apparent absence of measurable JAs in cold-treated WT and *fbs1–1* whole seedlings is reminiscent of the negligible JA levels found in mature unwounded plant tissues [52, 53]. Future studies could more thoroughly investigate JA levels in *fbs1–1* in the context of wounding stress, where differences in JA abundance between these genotypes might be amplified. Two observations, however, suggest that JA synthesis does occur in *fbs1–1*, at least on some level in specific situations. First, we note that only a subset of JA biosynthesis genes is under-expressed in *fbs1–1* (Fig. 4 and Additional file 4: Figure S4). At least two of the genes that are expressed at roughly equal levels between WT and *fbs1–1*, *LOX3* and *OPR3*, are themselves inducible by JA as part of a positive feedback effect to bolster a robust response [55]. If JA levels were decreased in *fbs1–1* then it might be expected that the *LOX3* and *OPR3* genes would also be under-expressed in this mutant like the six JA biosynthesis genes that have diminished expression (Fig. 4). Second, *Arabidopsis* JA biosynthesis mutants completely deficient in active JAs, such as the *fad3–2 fad7–2 fad8* triple mutant, *dad1*, *dde1*, and *opr3*, are all male sterile and do not set seed without exogenous application of JA [56–59]. The *fbs1–1* mutant used here is fertile and seed set in our growth conditions was identical to WT, indicating clearly that there are sufficient JA levels in male organs to fulfill reproductive requirements. Therefore, future studies could also probe tissue-specific differences of JA levels between *fbs1–1* and WT. Indeed, the JA biosynthesis gene *AOC1*, for which we measured a striking three orders of magnitude lower expression in *fbs1–1* seedlings compared to WT, is predominantly expressed in leaves [54]. It may be that loss of *FBS1* only affects JA levels in particular tissue types or at particular developmental stages.

Over nearly the last half century, the majority of work investigating JA has focused on its regulatory function in biotic stresses, where JA and related oxylipins are critical for responses to bacterial pathogens, such as *Pseudomonas syringae,* and many other fungi and insect pests [60]. However, recent studies show that JA also has regulatory roles in mitigating the effects of abiotic stresses [61–64]. JA regulates freezing tolerance controlled by the CBF pathway, where JAZ proteins physically interact with and inhibit ICE1 and ICE2 transcription factors to reduce expression of the cold regulon [62, 63]. Genetic analysis with various JA biosynthesis and response mutants has shown that JA is required for basal thermotolerance [61]. JA also enhances salt stress tolerance in wheat by increasing antioxidant levels in order to reduce the effects of ROS stimulated by high salinity conditions [64]. Because it is broadly induced by abiotic stresses and is co-expressed with critical regulators of abiotic stress responses, *FBS1* and its corresponding effects on JA processes might be important for directly alleviating the effects of abiotic stresses, such as cold, salt, drought, UV-B light, and heat (Fig. 1, Additional file 6: Table S1). However, some *FBS1* co-expressed genes have roles in biotic stresses (Additional file 6: Table S1), and some *FBS1*-dependent genes have roles in defense (Fig. 2). Therefore, the function of *FBS1* could be to help facilitate JA defense processes in abiotic stress conditions in order to protect compromised plant tissues from opportunistic pathogens and insect pests. It is also possible that *FBS1* acts, perhaps through modulation of JA processes, to abrogate a common issue resulting from both abiotic and biotic stresses, such as water deficit [65]. Future investigations with *fbs1–1* plants that have been challenged with various pests or pathogens, perhaps in combination with abiotic stresses, will help resolve these questions. Interestingly, the six under-expressed JA biosynthesis genes in *fbs1–1* were mis-expressed even in untreated seedlings at time 0 (Fig. 4), suggesting that *FBS1* might be required for a basal readiness that allows plants to deal with critical early stages of infection, attack, or onset of environmental stress.

### Interaction between *FBS1* and ABA-dependent genes

The interaction between *FBS1* and ABA processes appears to be more complex than the interaction between FBS1 and JA: ABA both induces and represses genes positively affected by *FBS1* in WT (Fig. 3a), but it is also the most potent inducer of genes that are negatively impacted by *FBS1* activity (Fig. 3b). Therefore, a simple model where SCF^FBS1^ regulates ABA biosynthesis or a core ABA pathway seems less likely. Specific genes more highly expressed in *fbs1–1* seedlings may provide clues as to how *FBS1* interacts with ABA-dependent processes. Among the genes more highly expressed in *fbs1–1* were five of twelve members of the *Arabidopsis* type I *LTP* gene family (Additional file 8: Table S3 and Additional file 10: Table S5). Multiple genetic studies using both loss-of-function mutants and over-expression lines have shown that *LTPs*, specifically type I *LTPs* in *Arabidopsis*, are required for dehydration-related abiotic stress responses, such as cold, drought, and salt [46, 47, 49]. Furthermore, *LTP3* and *LTP4* were recently shown to regulate ABA biosynthesis and stress responses [48, 50]. *FBS1* may therefore impose indirect restraint on downstream ABA responses by acting to reduce expression of *LTP* genes.

This up-regulation of some ABA genes in the absence of *FBS1*, concurrent with the down-regulation of set of JA genes, suggests that *FBS1* might act to balance these processes. In rice roots, ABA and JA antagonistically regulate expression of many salt-inducible genes belonging to different gene sets [66]. ABA suppresses many basal and JA-ethylene inducible defense genes, while ABA mutants show higher expression of these genes [67]. If the primary effects of *FBS1* on hormone signaling are positive for JA (Fig. 3a) and negative for at least one ABA module (Fig. 3b), then SCF^FBS1^ may act as a switch to control antagonistic interactions between these hormone pathways. Furthermore, if the JA genes observed here do function more in a defense capacity and the ABA genes in abiotic stresses, then SCF^FBS1^ may more broadly tailor a cellular response that is appropriate for either abiotic or biotic stresses.

### Mechanistic link between FBS1 and stress gene expression

FBS1 interacts with Arabidopsis Skp1 (ASK1) by yeast two-hybrid assays, indicating that it forms a canonical SCF complex capable of labeling proteins for degradation [68]. Understanding then exactly how FBS1 accomplishes its regulatory effects, and why it appears to regulate only a subset of JA biosynthesis genes, will likely come from identifying its ubiquitylation target(s). One possibility is that expression of this JA gene subset (Fig. 4) requires SCF^FBS1^-stimulated turnover of some unknown negative regulator of transcription, perhaps analogous to AUX/IAA or JAZ proteins [9, 32, 69]. In this scenario, the hypothetical negative regulator is not degraded in *fbs1–1* mutants and the effect is under-expression of this JA gene subset even in non-inducing conditions. It may be that this subset is supposed to selectively respond to abiotic stresses through FBS1 action stimulated by particular environmental conditions. FBS1 also interacts with five of 13 14–3-3 proteins encoded by the *Arabidopsis* genome in yeast two-hybrid or pull down assays [68]. Intriguingly, three of these FBS1 14–3-3 interactors have recently been shown to negatively regulate plant responses to cold and salt [70–72]. However, functional and mechanistic links between these 14–3-3 proteins and FBS1 are not established, and preliminary findings suggest that 14–3-3 proteins might not be SCF^FBS1^ ubiquitylation targets [68].

### Future genomics experiments

Our transcriptome analysis has yielded important information regarding the effects of *FBS1* on hormone pathways that will guide further efforts at delineating *FBS1* function. Because we analyzed publicly available array datasets from independent sources as part of this study [54, 73], however, our results should be interpreted with some caution [74]. A future more comprehensive RNA-sequencing (RNA-seq) analysis of the *FBS1*-dependent transcriptome could take multiple variables into consideration as part of a single experiment. First, because *FBS1* is broadly induced across many adverse conditions (Fig. 1), future analyses should use multiple stresses to determine whether there is a common set of *FBS1*-dependent differentially expressed genes in all stresses. Second, the stress-induction profile of *FBS1* is not identical between roots and shoots (Fig. 1b), and future transcriptomic investigations should take into consideration organ-specific outcomes. This will help establish more broadly how SCF^FBS1^ plays a role in whole plant stress physiology.

## Conclusions

While plant genomes are enriched in F-box genes, the vast majority of plant F-box proteins are unstudied and have unknown functions. Based on expression analysis, this work shows that F-box gene *FBS1* belongs to a wider set of established stress genes, which have significantly matching stress induction profiles across a broad range of unfavorable conditions. *FBS1* co-expressed genes include essential transcriptional regulators of both abiotic and biotic stress responses, a finding which implies that *FBS1* has a central role in these inducible stress responses as well. Our results show that *FBS1* is required for proper expression of known abiotic and biotic stress genes that are important for survival in adverse environments, and we therefore conclude that *FBS1* encodes a stress response regulator. Specifically, SCF^FBS1^ positively regulates some JA processes but seemingly only impacts a subset of JA related genes. *FBS1* also influences ABA processes, but *FBS1* has both positive and negative effects on ABA-inducible and -repressible genes. However, one noteworthy result of *FBS1* activity on ABA stress processes is its inhibitory effect on multiple members of the type I *LTP* gene family. LTP family members have numerous roles in stimulating ABA biosynthesis and dehydration-related protective effects. In this context then, *FBS1* activity alters the balance between JA and at least one ABA stress gene network, and this model yields important hypotheses that will guide future efforts aimed at understanding crosstalk between these two indispensable stress hormones.

## Methods

### Analysis of publically available AtGenExpress microarray data

All public data sets used in this study were from Arabidopsis AtGenExpress [55, 73, 75]. AtGenExpress is a large-scale project to produce genome-wide expression profiles encompassing different developmental time points and tissues, in response to numerous chemicals and hormones, and from plants treated with a multitude of biotic and abiotic stresses. These MIAME-compliant Affymetrix ATH1 data sets were produced by numerous international groups from plants grown and treated under strictly defined standard conditions, and they were designed to have high reproducibility with the intent that they would be used by the Arabidopsis community at large for cross-experiment comparisons and other meta-analyses. All arrays for abiotic stress data sets were scanned with a GeneChip Scanner 3000 and were normalized with global scaling [73]. Arrays for hormone treatments were scanned with an Affymetrix Gene Array Scanner 2500A and were also normalized with global scaling [55]. Some differences between the plants in our experiments and the plants used in the AtGenExpress data sets exist (ie. 12 vs. 16–18 day old plants), and we have noted in the text or legends these differences. Affymetrix ATH1 array .CEL files encompassing time points from 15 min to 24 h after treatments with heat, drought, and cold, as well as corresponding controls (Additional file 12: Table S7) were downloaded from The *Arabidopsis* Information Resource (TAIR) (http://www.arabidopsis.org/). A .cdf file was obtained from NASC the European Arabidopsis Stock Centre (http://arabidopsis.info/). Chips were scaled to the median chip fluorescence intensity and values processed by the Invariant Set Normalization method using DNA-Chip Analyzer version 2010.1 [76]. A two-fold filtering criteria was applied to produce a list of candidate genes differentially expressed between the time 0 (control) data sets and time points after stress treatment, and also between each treated time point and its corresponding untreated control collected at the same time in the time course. Candidate stress responsive F-box genes were selected from these differentially expressed genes.

### Co-expression analysis and e-Northerns

Stress- and hormone-specific gene expression profiles in young *Arabidopsis* plants were investigated using the Expression Angler and e-Northern tools accessed via the web interface of the Bio-Analytic Resource (BAR) at the University of Toronto [22]. All data sets in this analysis were normalized to a target intensity value of 100. For co-expression analysis, the AtGenExpress Abiotic Stress Compendium was searched for probesets matching *FBS1* with a Pearson correlation co-efficient of *r* > 0.75; while for e-Northern analysis, the AtGenExpress Hormone Series was examined [55].

### Plant growth and treatment

An *Arabidopsis* line (RATM16–1777-1) harboring a transposon insertion in the first intron of *FBS1* (hereafter referred to as *fbs1–1*) and the corresponding No-0 wild-type line was obtained from RIKEN [77]. Seeds for all experiments were surface-sterilized in 50% bleach containing 0.01% Tween-20 and stratified for five days in 0.1% agarose in the dark at 4 °C. Seeds were then sown onto sterile Whatman filter paper (Whatman | GE Life Sciences, www.gelifescience.com) and placed on square plates containing 50 mL of MS medium at pH 5.8 with 0.8% type I micropropagation agar (Caisson Laboratories, www.caissonlabs.com). All seedlings were grown in Percival CU-30L2 growth chambers (Percival Scientific, www.percival-scientific.com), set to either constant 24 °C or 10 °C and identical long-day conditions (16 h light:8 h darkness) with cool white light at 100 μmol/m^2^/s. Seedlings for chilling treatments were grown for five days at 24 °C before being transferred at dawn of the sixth day to 10 °C and grown for seven additional days. Corresponding controls were left at 24 °C for the entire 12 days. For both microarray and qPCR, approximately 50 seedlings for each biological replicate were harvested, placed in 1.5 mL tubes, and immediately frozen in liquid nitrogen.

### RNA isolation

Frozen samples were pulverized in 1.5 mL tubes with micropestles. Total RNA was isolated with the Qiagen RNeasy Kit and contaminating genomic DNA digested on column with the Qiagen RNase-free DNase Set according to the manufacturer’s protocol (Qiagen, www.qiagen.com/us/).

### Microarray processing and analysis

Three biological replicates for both No-0 and *fbs1–1* were processed and run on the ATH1 array at the Institute for Integrative Genome Biology at the University of California Riverside (http://genomics.ucr.edu/facility/genomics.html) according to standard Affymetrix protocols (Affymetrix | Thermo Fisher Scientific, www.thermofisher.com). The .CEL files were analyzed in R using the Bioconductor package RankProd [78]. Prior to processing, chips were normalized in R using Robust Multi-Array (RMA) normalization. Pearson correlation co-efficient (*r*) values between all independent biological replicate comparisons of the same genotype were above 0.988 (Additional file 11: Table S8). Probe set signal intensities from *fbs1–1* samples were compared to signal intensities from No-0 samples using a Percentage of False Prediction cut-off of 0.05.

### AgriGO

Gene Ontology analysis was assessed with agriGO (http://bioinfo.cau.edu.cn/agriGO/) [79]. Singular Enrichment Analysis was performed for *Arabidopsis thaliana* against the Affymetrix ATH1 Genome Array (GPL198) reference. Significance was assessed using a Fisher test with the Yeutieli multiple hypothesis testing correction (False Discovery Rate under dependency) at a 0.05 significance level. The minimum number of mapping entries was set to five (Additional file 12: Table S6 and Additional file 13: Table S7).

### qPCR

First-strand cDNA was prepared from 1 μg of total RNA with the Maxima Universal First Strand cDNA Synthesis kit (Thermo Fisher Scientific, www.thermofisher.com/) and diluted 1:4 in RNase-free water prior to use. Transcript levels were determined with qPCR using Bio-Rad SsoAdvanced Universal Supermix and the CFX96 Real-Time PCR Detection System according to manufacturer’s protocols (Bio-Rad, www.bio-rad.com). Transcript levels of target genes were assessed using normalized relative quantity (NRQ) calculated from the Pfaffl method using the geometric mean of two reference gene quantities [80, 81]. Reference genes were *PP2A* (At1g13320) and *IPP2* (At3g02780) [82]. Primer sequences for all reference and target genes are in Additional file 8: Table S3.

### Quantification of JA and JA-Ile levels

Samples were extracted and analyzed using a method similar to that described in Chung et al. [52]. Briefly, analytes were extracted from ~500 mg of whole seedlings with ethyl acetate, dried under a stream of nitrogen and reconstituted in 70% MeOH. Diydro-JA and C^13^_6_ JA-Ile were used as internal standards for JA and JA-Ile, respectively. Samples were analyzed on a Waters Xevo G2 QTOF equipped with an Acquity UPLC (Waters). A BEH C18 column (1.7 um, 2.1 × 50 mm, Waters) was used for chromatography with a gradient of (A) 0.5% formic acid and (B) methanol, applied at a 0.4 mL/min flow rate. Mass spectra were recorded in negative ion mode. Transitions were monitored for JA (m/z 209-- > 59), dihydroJA (m/z 211-- > 59), JA-Ile (m/z 322-- > 130), and 13C6-JA-Ile (m/z 328-- > 136) using a 28-V collision cell energy.

## Additional files

Additional file 1: Figure S1. qPCR analysis of *FBS1* expression. Seven day-old *Arabidopsis* wild type (Col-0) either untreated (time 0) or treated for up to three hours with 37 °C. The average relative quantity (RQ) (± SEM) of transcript is shown from three independent experimental replicates. Samples are normalized to *PP2A* within the same sample and to the wild type expression level for that gene in untreated seedlings. (PNG 35 kb)

Additional file 2: Figure S2. qPCR analysis of response to 10 °C. Seven day-old *Arabidopsis* wild type (white bars) and *fbs1–1* (black bars) seedlings were either untreated (time 0) or treated for up to three hours with 10 °C chilling temperature Transcript abundances for **(A)** *CBF1*, **(B)** *CBF2*, **(C)** *CBF3*, and **(D)** *FBS1* are shown. All four genes and both genotypes have statistically significant differences between the time 0 untreated and both treated time point samples, but no statistically significant differences between the genotypes (two-way ANOVA, *p* < 0.05). Shown are the average relative quantities (NRQs) (± SEM) normalized to *IPP2* and *PP2A* within the same sample and to the wild type expression level for that gene in untreated seedlings, which was set to 1, from three independent experimental replicates. (TIFF 18218 kb)

Additional file 3: Figure S3. Hierarchical clustering of expression patterns in 121 AtGenExpress hormone datasets of 267 genes more highly expressed in wild type plants. Genes are hierarchically clustered on the y-axis according to expression profile similarity. For each treatment, the exposure time for the given chemical increases from left to right. (TIFF 10502 kb)

Additional file 4: Figure S4. qPCR analysis of non-differentially expressed JA biosynthetic genes. Seven day-old *Arabidopsis* wild type (white bars) and *fbs1–1* (black bars) seedlings were either untreated (time 0) or treated for up to three hours with 10 °C chilling temperature Transcript abundances for **(A)** *LOX1*, **(B)** *LOX3*, **(C)** *LOX4*, **(D)** *LOX5*, **(E)** *LOX6*, **(F)** *AOC4*, **(G)** *OPR2*, and **(H)** *OPR3* are shown. Shown are the average relative quantities (NRQs) (± SEM) normalized to *IPP2* and *PP2A* within the same sample and to the wild type expression level for that gene in untreated seedlings, which was set to 1, from three independent experimental replicates. (TIFF 20532 kb)

Additional file 5: Figure S5. Extracted ion chromatograms of Jasmonic Acid (JA) and dihydro-JA (IS). Chromatograms are shown for **(A)** a calibration standard with 50 pmol JA, 15 pmol IS, **(B)** WT 24 °C, **(C)** WT 10 °C, **(D)** *fbs1–1* 24 °C, and **(E)** *fbs1–1* 10 °C. All samples have 15 pmol internal standard (IS). The peak for jasmonic acid appears at 2.82 min in the standard but is absent in all samples. The peak for the IS appears at 2.99 min and is present in all standards and samples. (PNG 145 kb)

Additional file 6: Table S1. Genes co-expressed with *FBS1* in nine abiotic stresses in roots and shoots. The 39 genes significantly co-expressed with *FBS1* (*r* > 0.75) across 272 AtGenExpress ATH1 data sets are listed. Abbreviated annotations are based on the TAIR 10.0 genome and published experiments. Designation as a validated abiotic (“A”) or biotic (“B”) stress gene in this table required published experimental evidence (ie. phenotype in knockout line) beyond induction or repression of the gene by stress. Genes encoding transcription factors or other signal transduction components were designated “R” as regulators of stress responses [23–31, 33, 83–95]. (DOC 173 kb)

Additional file 7: Table S2. 267 genes more highly expressed in WT. (XLSX 64 kb)

Additional file 8: Table S3. 254 genes more highly expressed in *fbs1–1*. (XLSX 67 kb)

Additional file 9: Table S4. Genes in the response to JA stimulus GO category more highly expressed in 12 day-old *Arabidopsis* wild type (No-0) seedlings treated for seven days with a 10 °C chilling temperature. *Arabidopsis* Genome Initiative (AGI) numbers, common gene name, and an abbreviated annotation based on the TAIR10 genome are indicated in the table. (DOC 31 kb)

Additional file 10: Table S5. Genes in lipid localization and lipid transport categories more highly expressed in *fbs1–1* seedlings. (DOC 32 kb)

Additional file 11: Table S8. Pearson correlation coefficients between experimental replicates for probesets on ATH1 arrays. (DOC 33 kb)

Additional file 12: Table S6. qPCR primer sequences. (DOC 39 kb)

Additional file 13: Table S7. AtGenExpress data sets used in Fig. 1a and Table 1. (DOC 28 kb)

## Abbreviations

ABA: Abscisic acid
AOC: ALLENE OXIDE CYCLASE
AOS: ALLENE OXIDE SYNTHASE
ASK1: Arabidopsis Skp1
BAR: Bio-analytic resource
CBF: C-repeat binding factor
ERF: ETHYLENE RESPONSE FACTOR
FBS1: F-BOX STRESS INDUCED 1
GA: Gibberellic acid
GO: Gene ontology
IAA: Indole-3-acetic acid
JA: Jasmonic acid
JA-Ile: Jasmonate-isoleucine
JAZ: JASMONATE ZIM-DOMAIN
LC-MS: Liquid chromatography-mass spectrometry
LOX: LIPOXYGENASE
LTP: LIPID TRANSFER PROTEIN
MeJA: Methyl jasmonate
NRQ: Normalized relative quantity
OPR: 12-OXOPHYTODIENOIC ACID REDUCTASE
qPCR: Quantitative real-time polymerase chain reaction
RMA: Robust multi-array
RNA-seq: RNA-sequencing
ROS: Reactive oxygen species
RT-PCR: Reverse transcription polymerase chain reaction
SA: Salicylic acid
SCF: Skp1-Cullin-F-box
UPS: Ubiquitin 26S proteasome system
WT: Wild type
