# Supplementary figures and images for: Transcriptome analysis uncovers Arabidopsis F-BOX STRESS INDUCED 1 as a regulator of jasmonic acid and abscisic acid stress gene expression
Source: BMC Genomics. 2017 Jul 17;18:533. doi: 10.1186/s12864-017-3864-6 (PMC5512810; doi:10.1186/s12864-017-3864-6)

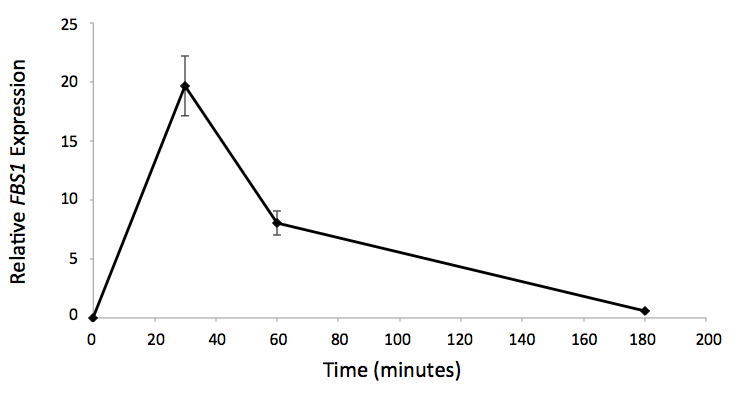

Supplement: Supplementary file 1 — qPCR analysis of FBS1 expression. Seven day-old Arabidopsis wild type (Col-0) either untreated (time 0) or treated for up to three hours with 37 °C. The average relative quantity (RQ) (± SEM) of transcript is shown from three independent experimental replicates. Samples are normalized to PP2A within the same sample and to the wild type expression level for that gene in untreated seedlings. (PNG 35 kb) [file 12864_2017_3864_MOESM1_ESM.png]

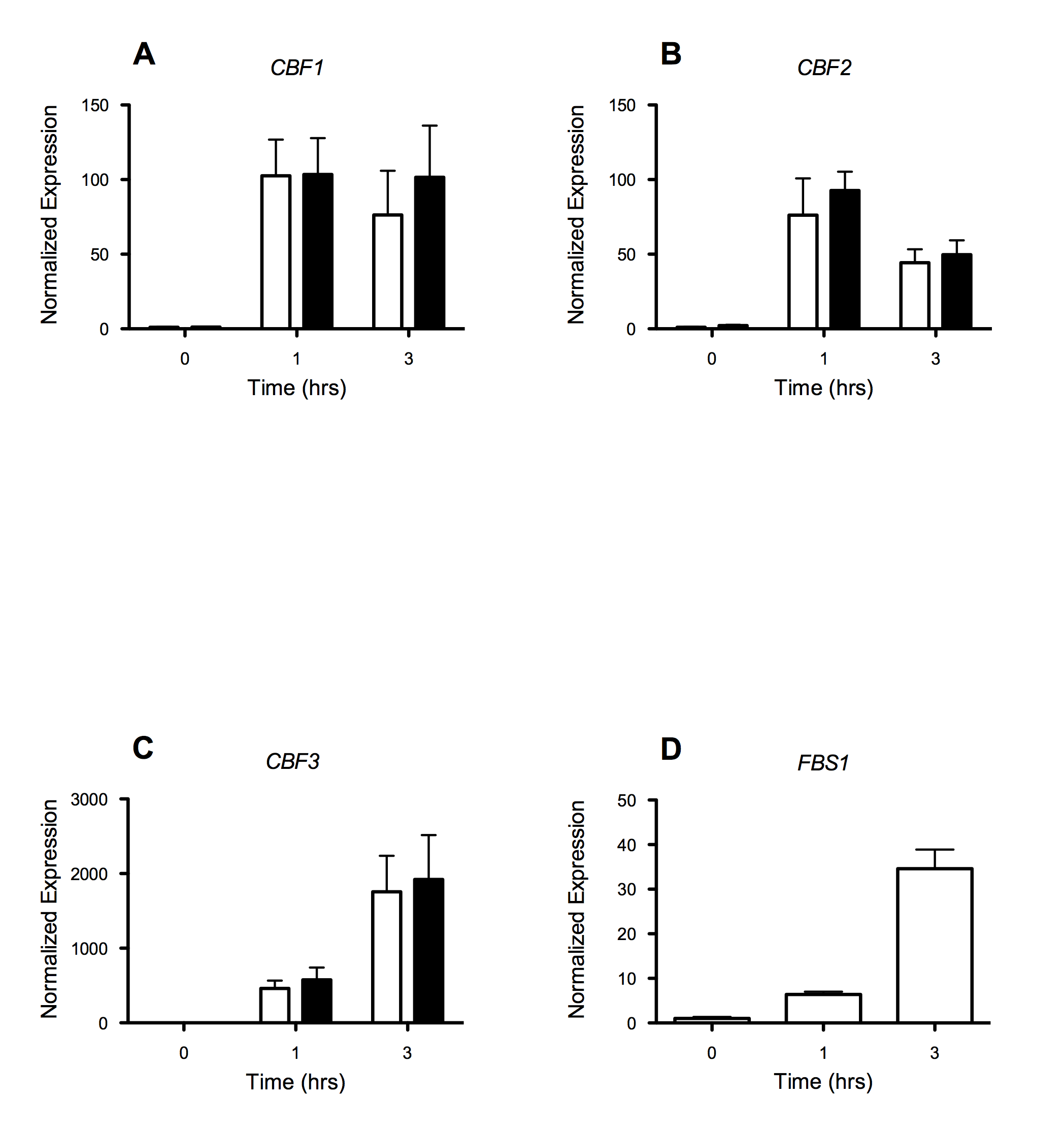

Supplement: Supplementary file 2 — qPCR analysis of response to 10 °C. Seven day-old Arabidopsis wild type (white bars) and fbs1–1 (black bars) seedlings were either untreated (time 0) or treated for up to three hours with 10 °C chilling temperature Transcript abundances for (A) CBF1, (B) CBF2, (C) CBF3, and (D) FBS1 are shown. All four genes and both genotypes have statistically significant differences between the time 0 untreated and both treated time point samples, but no statistically significant differences between the genotypes (two-way ANOVA, p < 0.05). Shown are the average relative quantities (NRQs) (± SEM) normalized to IPP2 and PP2A within the same sample and to the wild type expression level for that gene in untreated seedlings, which was set to 1, from three independent experimental replicates. (TIFF 18218 kb) [file 12864_2017_3864_MOESM2_ESM.tiff]

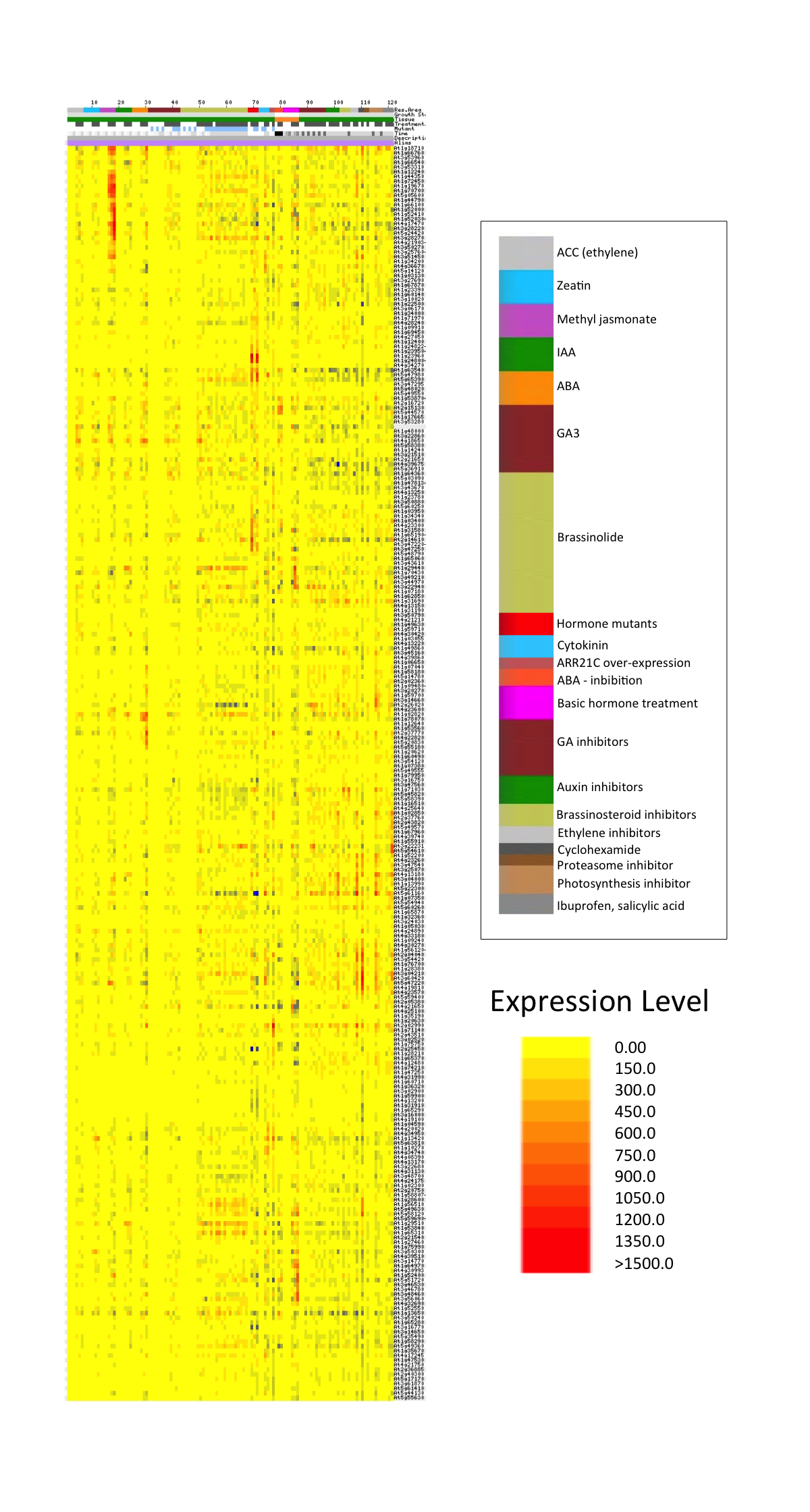

Supplement: Supplementary file 3 — Hierarchical clustering of expression patterns in 121 AtGenExpress hormone datasets of 267 genes more highly expressed in wild type plants. Genes are hierarchically clustered on the y-axis according to expression profile similarity. For each treatment, the exposure time for the given chemical increases from left to right. (TIFF 10502 kb) [file 12864_2017_3864_MOESM3_ESM.tif]

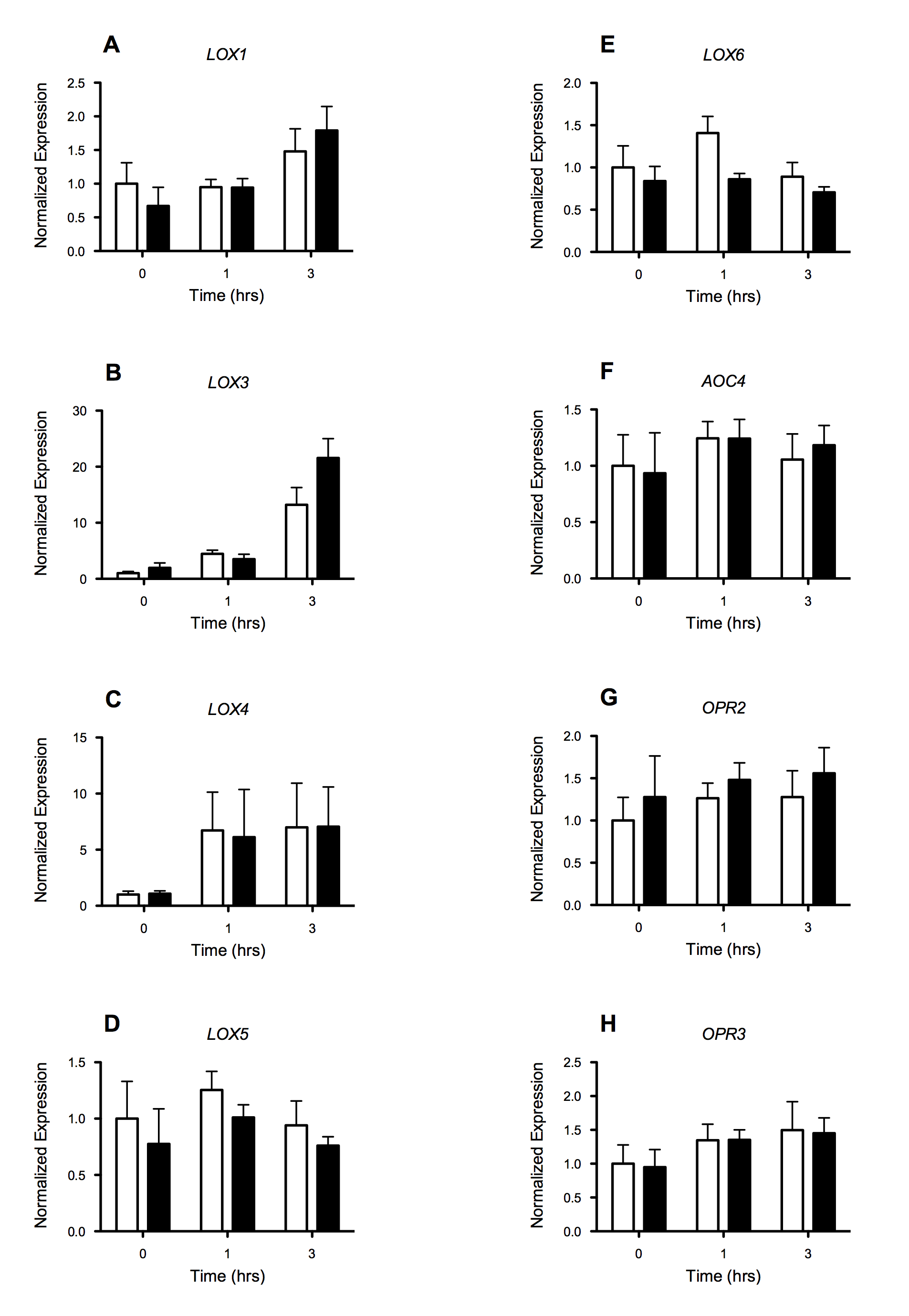

Supplement: Supplementary file 4 — qPCR analysis of non-differentially expressed JA biosynthetic genes. Seven day-old Arabidopsis wild type (white bars) and fbs1–1 (black bars) seedlings were either untreated (time 0) or treated for up to three hours with 10 °C chilling temperature Transcript abundances for (A) LOX1, (B) LOX3, (C) LOX4, (D) LOX5, (E) LOX6, (F) AOC4, (G) OPR2, and (H) OPR3 are shown. Shown are the average relative quantities (NRQs) (± SEM) normalized to IPP2 and PP2A within the same sample and to the wild type expression level for that gene in untreated seedlings, which was set to 1, from three independent experimental replicates. (TIFF 20532 kb) [file 12864_2017_3864_MOESM4_ESM.tiff]

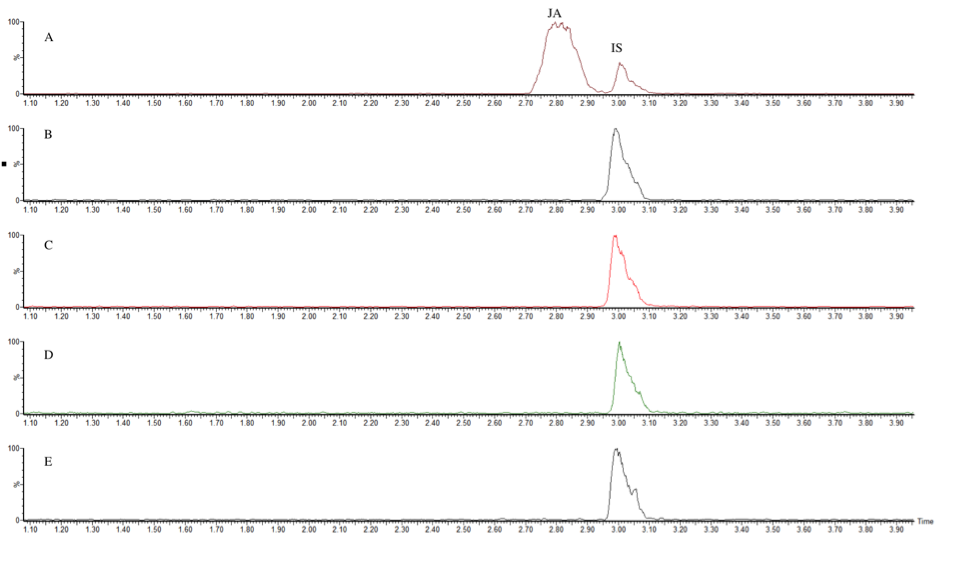

Supplement: Supplementary file 5 — Extracted ion chromatograms of Jasmonic Acid (JA) and dihydro-JA (IS). Chromatograms are shown for (A) a calibration standard with 50 pmol JA, 15 pmol IS, (B) WT 24 °C, (C) WT 10 °C, (D) fbs1–1 24 °C, and (E) fbs1–1 10 °C. All samples have 15 pmol internal standard (IS). The peak for jasmonic acid appears at 2.82 min in the standard but is absent in all samples. The peak for the IS appears at 2.99 min and is present in all standards and samples. (PNG 145 kb) [file 12864_2017_3864_MOESM5_ESM.png]
