# Supplementary material for: Transcriptome analysis uncovers Arabidopsis F-BOX STRESS INDUCED 1 as a regulator of jasmonic acid and abscisic acid stress gene expression
Source: BMC Genomics. 2017 Jul 17;18:533. doi: 10.1186/s12864-017-3864-6 (PMC5512810; doi:10.1186/s12864-017-3864-6)
Supplement: Supplementary file 6 — Genes co-expressed with FBS1 in nine abiotic stresses in roots and shoots. The 39 genes significantly co-expressed with FBS1 (r > 0.75) across 272 AtGenExpress ATH1 data sets are listed. Abbreviated annotations are based on the TAIR 10.0 genome and published experiments. Designation as a validated abiotic (“A”) or biotic (“B”) stress gene in this table required published experimental evidence (ie. phenotype in knockout line) beyond induction or repression of the gene by stress. Genes encoding transcription factors or other signal transduction components were designated “R” as regulators of stress responses [23–31, 33, 83–95]. (DOC 173 kb) [file 12864_2017_3864_MOESM6_ESM.doc]

**Table S1** **Genes co-expressed with *FBS1* in nine abiotic stresses in roots and shoots.** The 39 genes significantly co-expressed with *FBS1* (r > 0.75) across 272 AtGenExpress ATH1 data sets are listed. Abbreviated annotations are based on the TAIR 10.0 genome and published experiments. Designation as a validated abiotic (“A”) or biotic (“B”) stress gene in this table required published experimental evidence (ie. phenotype in knockout line) beyond induction or repression of the gene by stress. Genes encoding transcription factors or other signal transduction components were designated “R” as regulators of stress responses.

| **AGI Number** | **Common Name(s)** | **Abbreviated Annotation** | **Validation in Stress** | **Reference** |  |
| --- | --- | --- | --- | --- | --- |
| At1g27730 | *STZ, ZAT10* | Salt tolerance zinc finger, transcriptional repressor | A, R | [23, 24] |  |
| At1g29690 | *CAD1, NSL2* | Perforin domain-containing protein | B | [83] |  |
| At5g64660 | *CMPG2* | Functions in ubiquitin ligase, response to pathogen | B | [84] |  |
| At1g58420 |  | Uncharacterized conserved protein |  |  |  |
| At4g17615 | *CBL1* | Calcineurin B-like calcium sensor protein | A, R | [29] |  |
| At3g54000 |  | Uncharacterized conserved protein |  |  |  |
| At2g41640 |  | Glycosyltransferase |  |  |  |
| At3g49530 | *NAC062, NTL6* | Links cold signals and pathogen resistance responses | A, B, R | [30] |  |
| At3g46620 | *RDUF1* | C3HC4-type RING zinc finger family protein, ABA induced | A, R | [85] |  |
| At4g17490 | *ERF6* | Transcription factor, ROS and light stress | A, R | [86] |  |
| At2g41010 | *CAMBP25* | Calmodulin (CAM)-binding protein | A, R | [87] |  |
| At3g02840 |  | ARM repeat superfamily protein |  |  |  |
| At3g15210 | *ERF4* | Transcription factor, negatively regulates JA defense genes | B, R | [33] |  |
| At3g55980 | *SZF1* | Salt-inducible zinc finger, regulates salt genes | A, R | [24] |  |
| At5g06320 | *NHL3* | Similar to known disease associate proteins | B | [88] |  |
| At5g24590 | *NAC091* | Defense response to turnip crinkle virus | B, R | [89] |  |
| At3g56880 |  | VQ motif-containing protein |  |  |  |
| At5g28630 |  | Glycine-rich protein |  |  |  |
| At2g46400 | *WRKY46* | Transcription factor, acts in salt/osmotic stress, regulates ABA | A, R | [25] |  |
| At1g20823 | *ATL80* | RING/U-box superfamily protein, cold stress response | A | [90] |  |
| At4g29780 |  | Unknown protein |  |  |  |
| At4g18880 | *HSFA4A* | Regulates salt stress responses | A, R | [31] |  |
| At2g40140 | *SZF2, CZF2* | Zinc finger protein, regulates salt stress response | A, R | [24] |  |
| At5g11650 |  | Alpha/beta-hydrolases superfamily protein |  |  |  |
| At4g11280 | *ACS6* | Ethylene biosynthesis | A, B | [91, 92] |  |
| At3g28340 | *GATL10* | Galacturonosyltransferase-like 10 |  |  |  |
| At2g30040 | *MAPKKK14* | Mitogen-activated protein kinase kinase kinase 14 |  |  |  |
| At4g37260 | *MYB73* | Negative regulator of salt response | A, R | [28] |  |
| At2g21120 |  | Protein of unknown function |  |  |  |
| At1g19180 | *JAZ1* | Central negative regulator of jasmonic acid genes | B, R | [27] |  |
| At1g28370 | *ERF11* | Transcription factor, negative regulator of ethylene biosynthesis | A, R | [86] |  |
| At3g16720 | *ATL2* | RING-H2 protein, induced by chitin | B | [93] |  |
| At2g38470 | *WRKY33* | Transcription factor, negatively regulates defense pathways / ABA | B, R | [26] |  |
| At2g26530 |  | Protein of unknown function |  |  |  |
| At4g17230 | *SCL13* | Scarecrow-like protein, member of GRAS gene family. |  |  |  |
| At3g62260 |  | Protein phosphatase 2C family protein |  |  |  |
| At5g52050 | *DTX50* | Transporter, facilitates ABA sensitivity in drought | A | [94] |  |
| At2g35930 | *PUB23* | U-box E3 ligase, response to water stress and PAMP immunity | A, B, R | [95] |  |
| At5g54490 | *PBP1* | Putative EF-hand calcium binding protein |  |  |  |

References

23. Sakamoto H, et al. Arabidopsis Cys2/His2-type zinc-finger proteins function as transcription repressors under drought, cold, and high-salinity stress conditions. Plant Physiol. 2004;136(1):2734–46.

24. Sun J, et al. The CCCH-type zinc finger proteins AtSZF1 and AtSZF2 regulate salt stress responses in Arabidopsis. Plant Cell Physiol. 2007;48(8):1148–58.

25. Ding ZJ, et al. Transcription factor WRKY46 regulates osmotic stress responses and stomatal movement independently in Arabidopsis. Plant J. 2014;79(1):13–27.

26. Jiang Y, Deyholos MK. Functional characterization of Arabidopsis NaCl-inducible WRKY25 and WRKY33 transcription factors in abiotic stresses. Plant Mol Biol. 2009;69(1–2):91–105.

27. Valenzuela CE, et al. Salt stress response triggers activation of the jasmonate signaling pathway leading to inhibition of cell elongation in Arabidopsis primary root. J Exp Bot. 2016;67(14):4209–20.

28. Kim JN, Nguyen NH, Jeong CY, Nguyen NT, Hong SW, Lee H. Loss of the R2R3 MYB, AtMyb73, causes hyper-induction of the SOS1 and SOS3 genes in response to high salinity in Arabidopsis. J Plant Physiol. 2013;170(16):1461–5.

29. Cheong YH. CBL1, a calcium sensor that differentially regulates salt, drought, and cold responses in Arabidopsis. The Plant Cell Online. 2003;15(8):1833–45.

30. Seo PJ, Park CM. A membrane-bound NAC transcription factor as an integrator of biotic and abiotic stress signals. Plant Signal Behav. 2010;5(5):481–3.

31. Perez-Salamo I, et al. The heat shock factor A4A confers salt tolerance and is regulated by oxidative stress and the mitogen-activated protein kinases MPK3 and MPK6. Plant Physiol. 2014;165(1):319–34.

33. Yang Z, et al. Arabidopsis ERF4 is a transcriptional repressor capable of modulating ethylene and abscisic acid responses. Plant Mol Biol. 2005;58(4):585–96.

83. Asada Y, et al. The Arabidopsis NSL2 negatively controls systemic acquired resistance via hypersensitive response. Plant Biotechnology. 2011;28(1):9–15.

84. Heise A, et al. Two immediate-early pathogen-responsive members of the AtCMPG gene family in Arabidopsis Thaliana and the W-box-containing elicitor-response element of AtCMPG1. Proc Natl Acad Sci U S A. 2002;99(13):9049–54.

85. Li J, et al. The E3 ligase AtRDUF1 positively regulates salt stress responses in Arabidopsis Thaliana. PLoS One. 2013;8(8):e71078.

86. Dubois M, et al. The ETHYLENE RESPONSE FACTORs ERF6 and ERF11 antagonistically regulate Mannitol-induced growth inhibition in Arabidopsis. Plant Physiol. 2015;169(1):166–79.

87. Perruc E, et al. A novel calmodulin-binding protein functions as a negative regulator of osmotic stress tolerance in Arabidopsis Thaliana seedlings. Plant J. 2004;38(3):410–20.

88. Varet A. The Arabidopsis NHL3 Gene encodes a plasma membrane protein and its Overexpression correlates with increased resistance to Pseudomonas syringae pv. Tomato DC3000. Plant Physiol. 2003;132(4):2023–33.

89. Donze T, Qu F, Twigg P, Morris TJ. Turnip crinkle virus coat protein inhibits the basal immune response to virus invasion in Arabidopsis by binding to the NAC transcription factor TIP. Virology. 2014;449:207–14.

90. Suh JY, Kim WT. Arabidopsis RING E3 ubiquitin ligase AtATL80 is negatively involved in phosphate mobilization and cold stress response in sufficient phosphate growth conditions. Biochem Biophys Res Commun. 2015;463(4):793–9.

91. Datta R, et al. Glutathione regulates 1-Aminocyclopropane-1-Carboxylate Synthase transcription via WRKY33 and 1-Aminocyclopropane-1-Carboxylate Oxidase by modulating messenger RNA stability to induce ethylene synthesis during stress. Plant Physiol. 2015;169(4):2963–81. 1207

92. Gravino M, et al. Ethylene production in Botrytis Cinerea- and oligogalacturonide-induced immunity requires calcium-dependent protein kinases. Plant J. 2015;84(6):1073–86.

93. Serrano M, et al. Chemical interference of pathogen-associated molecular pattern-triggered immune responses in Arabidopsis reveals a potential role for fatty-acid synthase type II complex-derived lipid signals. J Biol Chem. 2007;282(9):6803–11.

94. Zhang H, et al. A DTX/MATE-type transporter facilitates abscisic acid efflux and modulates ABA sensitivity and drought tolerance in Arabidopsis. Mol Plant. 2014;7(10):1522–32. 1224

95. Seo DH, et al. Roles of four Arabidopsis U-box E3 ubiquitin ligases in negative regulation of abscisic acid-mediated drought stress responses. Plant Physiol. 2012;160(1):556–68.
