# Supplementary material for: Transcriptome analysis uncovers Arabidopsis F-BOX STRESS INDUCED 1 as a regulator of jasmonic acid and abscisic acid stress gene expression
Source: BMC Genomics. 2017 Jul 17;18:533. doi: 10.1186/s12864-017-3864-6 (PMC5512810; doi:10.1186/s12864-017-3864-6)
Supplement: Supplementary file 9 — Genes in the response to JA stimulus GO category more highly expressed in 12 day-old Arabidopsis wild type (No-0) seedlings treated for seven days with a 10 °C chilling temperature. Arabidopsis Genome Initiative (AGI) numbers, common gene name, and an abbreviated annotation based on the TAIR10 genome are indicated in the table. (DOC 31 kb) [file 12864_2017_3864_MOESM9_ESM.doc]

**Table S4** Genes in the response to JA stimulus GO category more highly expressed in 12 day-old *Arabidopsis* wild type (No-0) seedlings treated for seven days with a 10^o^C chilling temperature. *Arabidopsis* Genome Initiative (AGI) numbers, common gene name, and an abbreviated annotation based on the TAIR10 genome are indicated in the table.

| **AGI Number** | **Common Name** | **Abbreviated Annotation** |
| --- | --- | --- |
| At5g47220 | *ERF2* | transcription factor, positive regulator of JA defense genes |
| At1g72450 | *JAZ6* | negative central regulator of jasmonic acid genes; degraded in response to JA |
| At1g71030 | *MYBL2* | single repeat MYB transcription factor, negatively controls anthocyanin synthesis |
| At4g23600 | *CORI3* | Cystine lyase, generates precursors to ethylene biosynthesis |
| At1g70700 | *JAZ9* | Central negative regulator of jasmonic acid genes |
| At2g16720 | *MYB7* | R2R3-MYB transcription factor, negative regulation of flavonol biosynthesis |
| At1g18710 | *MYB47* | R2R3-MYB transcription factor |
| At2g02990 | *RNS1* | Ribonuclease, responds to inorganic phosphate starvation |
