# Supplementary material for: Transcriptome analysis uncovers Arabidopsis F-BOX STRESS INDUCED 1 as a regulator of jasmonic acid and abscisic acid stress gene expression
Source: BMC Genomics. 2017 Jul 17;18:533. doi: 10.1186/s12864-017-3864-6 (PMC5512810; doi:10.1186/s12864-017-3864-6)
Supplement: Supplementary file 10 — Genes in lipid localization and lipid transport categories more highly expressed in fbs1–1 seedlings. (DOC 32 kb) [file 12864_2017_3864_MOESM10_ESM.doc]

**Table S5** Genes in lipid localization and lipid transport categories more highly expressed in *fbs1-1* seedlings.

| **AGI Number** | **Common Name** | **Abbreviated Annotation** |
| --- | --- | --- |
| At2g38530 | *LTP2* | Lipid transfer protein |
| At5g59310 | *LTP4* | Lipid transfer protein |
| At3g08770 | *LTP6* | Lipid transfer protein |
| At2g15050 | *LTP7* | Lipid transfer protein |
| At2g13820 | *XYP2* | Bifunctional inhibitor/lipid-transfer protein/seed storage 2S albumin superfamily |
| At5g64080 | *XYP1* | Bifunctional inhibitor/lipid-transfer protein/seed storage 2S albumin superfamily |
| At3g53980 |  | Bifunctional inhibitor/lipid-transfer protein/seed storage 2S albumin superfamily |
| At5g05960 |  | Bifunctional inhibitor/lipid-transfer protein/seed storage 2S albumin superfamily |
| At4g33550 |  | Bifunctional inhibitor/lipid-transfer protein/seed storage 2S albumin superfamily |
| At3g18280 |  | Bifunctional inhibitor/lipid-transfer protein/seed storage 2S albumin superfamily |
