# Supplementary material for: Transcriptome analysis uncovers Arabidopsis F-BOX STRESS INDUCED 1 as a regulator of jasmonic acid and abscisic acid stress gene expression
Source: BMC Genomics. 2017 Jul 17;18:533. doi: 10.1186/s12864-017-3864-6 (PMC5512810; doi:10.1186/s12864-017-3864-6)
Supplement: Supplementary file 11 — Pearson correlation coefficients between experimental replicates for probesets on ATH1 arrays. (DOC 33 kb) [file 12864_2017_3864_MOESM11_ESM.doc]

**Table S8** Pearson correlation coefficients between experimental replicates for probesets on ATH1 arrays

|  | **WT_A (43)** | **WT_B (59)** | **WT_C (60)** | ***fbs1-1*_A (45)** | ***fbs1-1*_B (61)** | ***fbs1-1*_C (62)** |
| --- | --- | --- | --- | --- | --- | --- |
| **WT_A (43)** | 1.00000 |  |  |  |  |  |
| **WT_B (59)** | 0.98867 | 1.00000 |  |  |  |  |
| **WT_C (60)** | 0.98867 | 0.99582 | 1.00000 |  |  |  |
| ***fbs1-1*_A (45)** | 0.98438 | 0.98587 | 0.98422 | 1.00000 |  |  |
| ***fbs1-1*_B (61)** | 0.98068 | 0.98856 | 0.98722 | 0.99116 | 1.00000 |  |
| ***fbs1-1*_C (62)** | 0.97986 | 0.98757 | 0.98623 | 0.99193 | 0.99511 | 1.00000 |
