# Supplementary material for: Transcriptome analysis uncovers Arabidopsis F-BOX STRESS INDUCED 1 as a regulator of jasmonic acid and abscisic acid stress gene expression
Source: BMC Genomics. 2017 Jul 17;18:533. doi: 10.1186/s12864-017-3864-6 (PMC5512810; doi:10.1186/s12864-017-3864-6)
Supplement: Supplementary file 12 — qPCR primer sequences. (DOC 39 kb) [file 12864_2017_3864_MOESM12_ESM.doc]

**Table S6** qPCR primer sequences

| **Common Name** | **AGI Number** | **Forward Primer** | **Reverse Primer** |
| --- | --- | --- | --- |
| *LOX1* | At1g55020 | ATGCAAGCAGGACACTTCTG | GCGAGTCGTAGACACCTTCA |
| *LOX2* | At3g45140 | AACTACGATTGCATGGGTCA | TCGGTTGGGAAAGTATCCTC |
| *LOX3* | At1g17420 | CGACCTCGTGTCGATTCTAA | CGTAGCCACCGTAAGGGTAT |
| *LOX4* | At1g72520 | CTTCAACCGACACCGATATG | CTGCTCATCTCTTGGCACAT |
| *LOX5* | At3g22400 | AAGCTCCGTGATGTTATCCC | CTCTTTGAGGATGTCAGGCA |
| *LOX6* | At1g67560 | AACCGACCTACCCTGTTGAG | CTTGGAGCTGTGTCGGTAGA |
| *AOC1* | At3g25760 | TCCTCCTCTCCGACAAGTTT | TTGAACTTTGCTTGGTCTGG |
| *AOC2* | At3g25770 | GTACCGCCGTCTAAGGACAT | TAACTCCGCTAGGCTCCAGT |
| *AOC3* | At3g25780 | GGCTCTGGAATCTTTGAAGG | AAGCTCCAGCGGTAAATCAT |
| *AOC4* | At1g13280 | CGAGGACACGTTTCTTGCTA | GCTCCACAGGTAAATCAGCA |
| *AOS* | At5g42650 | GTCATCTCGAAACCGGATCT | CGAGCCAAGAAATTAAAGGC |
| *OPR1* | At1g76680 | GTCGATGCAGTTGCTAAGGA | TGTTCAAAGATTCCGCCATA |
| *OPR2* | At1g76690 | TAGGGCTGTACATGGTGGAA | CCCTCATTGGCATTAGTGTG |
| *OPR3* | At2g06050 | CGTCAACGAACAAACCAATC | GAATCGCATTCAAAGCAGAA |
| *FBS1* | At1g61340 | GGCAGATTTGGTTGGGATAA | TTGCGGTTTATCCTCGAAAG |
| *CBF1* | At4g25490 | GGAGACAATGTTTGGGATGC | CGACTATCGAATATTAGTAACTCCAAAGCGACACG |
| *CBF2* | At4g25470 | GGATGCTCATGGTCTTGACAT | TCTTCATCCATATAAAACGCATCTTG |
| *CBF3* | At4g25480 | GGCGTTTCAGGATGAGATGT | CAACAAACTCGGCATCTCAA |
| *IPP2* | At3g02780 | GTATGAGTTGCTTCTCCAGCAAAG | GAGGATGGCTGCAACAAGTGT |
| *PP2A* | At1g13320 | TAACGTGGCCAAAATGATGC | GTTCTCCACAACCGCTTGGT |
