# Supplementary material for: Transcriptome analysis uncovers Arabidopsis F-BOX STRESS INDUCED 1 as a regulator of jasmonic acid and abscisic acid stress gene expression
Source: BMC Genomics. 2017 Jul 17;18:533. doi: 10.1186/s12864-017-3864-6 (PMC5512810; doi:10.1186/s12864-017-3864-6)
Supplement: Supplementary file 13 — AtGenExpress data sets used in Fig. 1a and Table 1. (DOC 28 kb) [file 12864_2017_3864_MOESM13_ESM.doc]

**Table S7** AtGenExpress data sets used in Figure 1A and Table 1

| **Array Set Series** | **Submission Number** | **TAIR Accession** |
| --- | --- | --- |
| Cold stress time course | ME00325 | ExpressionSet:1007966553 |
| Drought stress time course | ME00338 | ExpressionSet:1007966668 |
| Heat stress time course | ME00339 | ExpressionSet:1007967124 |
| Methyl jasmonate time course | ME00337 | ExpressionSet:1007965964 |
